# Disseminated cryptococcosis and active pulmonary tuberculosis co-infection in an otherwise healthy adult

**Published:** 2015-07-06

**Authors:** Ghaemeh Nabaei, Shirin Afhami

**Affiliations:** 1 Iranian Center of Neurological Research AND Department of Neurology, School of Medicine, Shariati Hospital, Tehran University of Medical Sciences Tehran, Iran; 2 Department of Infectious Disease, School of Medicine, Shariati Hospital, Tehran University of Medical Sciences, Tehran, Iran

**Keywords:** Cryptococcosis, Pulmonary Tuberculosis, Coinfection

## Introduction

Co-infection with Mycobacterium tuberculosis (MTB) and Cryptococcus is very rare in immunocompetent hosts,^1^^,^^2^ and it is even more infrequent that the opportunistic yeast becomes disseminated in the presence of normal immune system and negative HIV test.^3^ We present an extremely uncommon case of severe fatal pulmonary and meningeal cryptococcosis along with fungemia associated with active pulmonary TB in a 70-year-old Afghan man with normal CD4 and CD8 lymphocyte counts and negative HIV antibody test.

**Figure 1:**
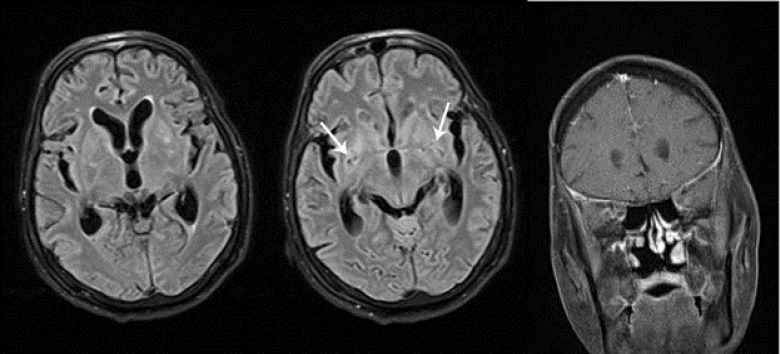
Brain magnetic resonance imaging (MRI) of the patient with cryptococcal infection shows bilateral enlarged perivascular spaces (white arrows) and basal ganglia hyperintensities in fluid-attenuated inversion recovery MRI along with bilateral mild basal leptomeningeal enhancement in contrast-enhanced MRI

**Figure 2:**
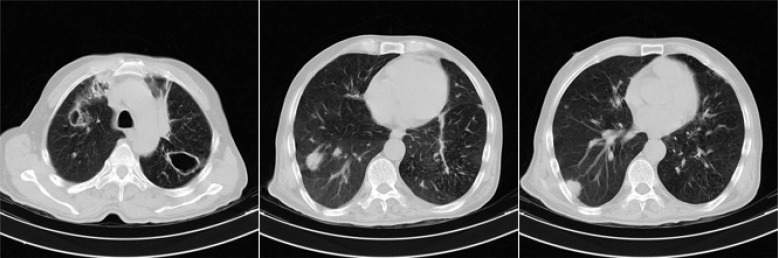
Chest computed tomography scan of our patient with positive bronchoalveolar lavage smear for tuberculosis and Cryptococcus neoformans show cavitations in both upper lobes along with bilateral multiple nodules

The patient was a 70-year-old Afghan man who had been immigrated to Iran 15 years ago, with an unremarkable medical history who was an active construction worker prior to initiation of his symptoms. He was admitted to hospital due to 40 days history of headache, weight loss, fatigue, anorexia, and occasional fever which was deteriorated in the last 10 days with superimposition of gait impairment, blurred vision, nausea and vomiting, mental confusion and memory loss. He didn’t have any significant cough or expectorations. His family history was unremarkable and he did not mention any known exposures to TB. In the physical examination, he was afebrile, drowsy, and had mild neck stiffness. Cranial nerves were normal, deep tendon reflexes were absent with downward plantar reflexes and despite normal muscle forces he couldn’t walk normally. Brain computed tomography (CT) scan without contrast showed communicating hydrocephalus and the brain magnetic resonance imaging (MRI) revealed slight degrees of basal meningitis followed by enlarged perivascular spaces and basal ganglia involvement in subsequent imagings (Figure 1). Cerebrospinal fluid (CSF) parameters were as follows: Opening pressure of 30 cm H_2_O; total white blood cell count of 495 cell/µl with 76% polymorphonuclear; protein level of 100 mg/dl; and a glucose level of 21 mg/dl. Chest CT scan showed multiple nodules and upper lobe cavities in both lungs (Figure 2). Although MTB-polymerase chain reaction and direct smear of CSF for mycobacteria were negative and adenosine deaminase activity level was low (1.5 U/l), CSF direct examination with Indian ink showed budding yeast cells with capsules compatible with Cryptococcus neoformans and CSF and blood culture were positive for the same element. The diagnosis of concomitant TB infection and cryptococcosis was made following a bronchoscopy, when bronchial alveolar lavage specimens were highly positive for acid-fast bacilli along with fungal elements and the sputum smears were positive for mycobacteria for 3 times. While finding the co-existence of these 2 infections made us highly suspicious of positive human immunodeficiency virus (HIV) test or impaired immunity, the patient’s HIV test was negative and flow cytometric findings showed normal CD4 and CD8 cell counts.

At first the patient was treated with a combination of anti-TB [isoniazid (300 mg daily), rifampin (600 mg daily), pyrazinamide (1500 mg daily) and ethambutol (1200 mg daily)] and steroid. When C. neoformans was detected on CSF culture, treatment with conventional amphotericin B was started because flucytosine and liposomal amphotericin B are not available in our country. After initiation of therapy, the patient was afebrile and his level of consciousness was improved, but 3 weeks later, he developed fever, drowsiness, and dyspnea. Further, CSF exam showed pleocytosis (with lymphocyte predominance), very low glucose level (< 10 mg/dl) and positive fungal smear and culture, and blood culture was persistently positive for the yeast. Despite treatment, the patient’s general condition was deteriorated and he eventually expired due to pulmonary complications, although investigations showed no signs of superimposed pulmonary thromboembolism or nosocomial infection.

The present case report describes the exceptional co-occurrence of disseminated cryptococcosis and active pulmonary TB in an otherwise healthy adult. This co-infection is almost always indicative of compromised cell-mediated immunity. Thus, its occurrence is very rare in immunocompetent individuals.^1^^,^^4^

Considering that disseminated cryptococcosis-defined by a positive culture from at least two different sites or a positive blood culture - is also a rare entity in healthy individuals.^3^ our unique adult case presents all of these rarities.

Up to now several cases of TB-cryptococcosis co-infection has been reported, but based on our knowledge there is only one other similar case which describes concurrence of severe infection with Cryptococcus gattii and MTB (central nervous system and pulmonary involvement) without positive fungal blood culture in an otherwise healthy 18 years old university student and without any evidence of CD4 cell count disturbance.^1^ In 2 other reports which have described the concomitant occurrence of pulmonary and meningeal tuberculosis (TB) along with meningeal cryptococcosis in a young otherwise healthy student^2^ and C. neoformans meningitis in a HIV-negative miliary TB-suspected patient,^5^ some degrees of CD4 cell count alteration was mentioned.

Patient’s treatment failure firstly can be due to his delay in referring to hospital, as although his symptoms had been emerged 40 days before admission he referred when the signs related to superimposed hydrocephalus was developed, and secondly because of unavailability of effective antifungal drugs (liposomal amphotericin B and flucytosine) in our country.

In conclusion, to prevent delay in diagnosis and initiation of therapy, the present case report emphasizes the importance of taking into account more than one infection occurring simultaneously in patients without significant comorbidities or immunodeficiency. 

## Notes:

**How to cite this article: **Nabaei G, Afhami Sh. Disseminated cryptococcosis and active pulmonary tuberculosis co-infection in an otherwise healthy adult. Iran J Neurol 2015; 14(3): 174-6.
